# Determination of Embryotoxic Effects of In Ovo Administered Propofol on Peripheral Blood Alpha Naphthyl Acetate Esterase and Acid Phosphatase-Positive Lymphocytes

**DOI:** 10.5152/TJAR.2023.22726

**Published:** 2023-02-01

**Authors:** Murat İzgi, Emrah Sur, Yasemin Öznurlu, Tuğba Özaydın, Tansu Kuşat

**Affiliations:** 1 Department of Anaesthesiology and Reanimation, Hacettepe University Faculty of Medicine, Ankara, Turkey; 2 Department of Histology and Embryology, Selçuk University Faculty of Veterinary, Konya, Turkey; 3 Department of Histology and Embryology, Selçuk University Health Sciences Institute, Konya, Turkey

**Keywords:** Alpha naphthyl acetate esterase, acid phosphatase, chicken embryo, enzyme histochemical methods, propofol

## Abstract

**Objective:**

The aim of this study is to determine the possible embryotoxic effects of propofol, a general anaesthetic agent that is commonly used in clinical practice, on peripheral blood lymphocytes using enzyme histochemical techniques.

**Methods:**

For this purpose, 430 laying hen fertile eggs were used for this study. The eggs were divided into 5 groups as control, solvent-control (saline), 2.5 mg kg^−1^ propofol, 12.5 mg kg^−1^ propofol, and 37.5 mg kg^−1^ propofol, and injections were performed via the air sac just before the incubation. The peripheral blood alpha naphthyl acetate esterase and acid phosphatase-positive lymphocyte ratios were determined on the hatching day.

**Results:**

No statistically significant difference was found between both alpha naphthyl acetate esterase and acid phosphatase-positive lymphocyte ratios of the control and solvent-control groups. However, when compared with the control and solvent-control groups, statistically significant decreases were observed in the peripheral blood alpha naphthyl acetate esterase and acid phosphatase-positive lymphocyte ratios of the chicks from the propofol-injected groups. Besides, the difference between 2.5 mg kg^−1^ and 12.5 mg kg^−1^ propofol groups is not significant, whereas the difference between these 2 groups and the 37.5 mg kg^−1^ propofol group was statistically significant (*P* < .05).

**Conclusions:**

It was concluded that propofol given to fertilised chicken eggs just before incubation caused significant decreases in both the peripheral blood alpha naphthyl acetate esterase and acid phosphatase-positive lymphocyte ratios.

**Main Points**

- The study aimed to determine the possible embryotoxic effects of propofol on peripheral blood lymphocytes.
- Three different doses of propofol were injected into the fertilised chicken eggs and the results were compared.
- Propofol given to fertilised chicken eggs just before incubation caused significant decreases in both the peripheral blood alpha naphthyl acetate esterase and acid phosphatase-positive lymphocyte ratios.

## Introduction

The effects of anaesthetic substances on the immune system have been one of the biggest problems of anaesthetists and surgeons for a long time. There is a consensus that most of the anaesthetic agents used in clinical practice adversely affect immune system cells, especially peripheral blood lymphocytes. It has been suggested that one of the important reasons underlying the recurrence of surgically excised tumours, especially in some types of cancer, may be the suppressive effects of anaesthetic agents on the immune system.^1,2^ In addition, it has been reported that anaesthetics used during surgical procedures affect the anti-oxidant system and lead to lymphocyte apoptosis, and as a result, they cause post-operative lymphocytopenia.^3^ In a previous study, Jakobsson et al^4^ investigated the effects of surgical procedure, radiotherapy application, radiotherapy, and cytotoxic drug combination on alpha naphthyl acetate esterase (ANAE)-positive cell counts on lung cancer patients and found that the surgical procedure caused a temporary decrease in ANAE-positive lymphocyte count.

Propofol, which has sedative, hypnotic, and antiemetic properties, is a short-acting intravenous (IV) induction agent that is frequently preferred for the purpose of general anaesthesia. Propofol, which has a rapid onset effect, no significant accumulation observed in its maintenance, and provides rapid recovery, is frequently preferred not only in human medicine but also in veterinary clinics for pet animals^5^ and chickens.^6^

Although previous studies are showing that propofol can be used safely when compared with other anaesthetic agents,^7,8^ in a study,^9^ desflurane and propofol anaesthesia were compared in patients with breast cancer and it was shown that the decrease in total leukocyte, lymphocyte, and natural killer cell counts in the first hour following the induction of anaesthesia was more pronounced in patients who underwent propofol anaesthesia. Yuki et al^10^ also showed that propofol suppressed T lymphocyte proliferation by inhibiting the communication between “lymphocyte function-related antigen-1” and intercellular adhesion molecules in vitro.

According to the US Federal Food and Drug Administration, propofol in pregnancy is in category B for which there is no clinical evidence in humans, most previous studies aimed at revealing the effects on exposed individuals. Studies on the possible embryotoxic or teratogenic effects of the substance in question are limited. However, it has been reported that the transplacental passage of propofol, which is a low molecular weight, fat-soluble, and nonionic substance, is quite rapid.^11^ This raises the concern that exposure to propofol at any time during pregnancy may cause serious problems in the embryo and/or newborn. Before newly developed drugs and chemicals are used in human and veterinary medicine, it is necessary to carry out detailed tests to reveal possible toxicity and side effects. An important advantage is that these tests are fast, low-cost, and repeatable. The Chicken Embryotoxicity Screening Test (CHEST), described by Jelinek^12^ and using fertile chicken eggs, made significant contributions to embryotoxicity and teratogenicity studies in this field. The first 72 hours of embryo development in fertile chicken eggs is very important for the next process of development. For this reason, it is reported that the applications should be carried out in the first 72 hours to see the obvious effects of chemical substances with teratogenic effects.^13^ Since the human embryo corresponds to the 2- to 8-week period when the human embryo is most sensitive to chemical and physical factors, injections into the eggs are recommended in this first 3-day period in embryotoxicity studies.^14^

Alpha-naphthyl acetate esterase enzyme is a lysosomal enzyme used to distinguish T lymphocyte, B lymphocyte, and monocytes from each other in tissue sections and peripheral blood smears of human and some animal species. It is suggested that the enzyme, which is thought to be involved in the cytotoxic functions of activated T lymphocytes and the removal of materials phagocytosed by macrophages, is acquired during the maturation process of T lymphocytes.^15^

It is suggested that acid phosphatase (ACP-ase), a lysosomal enzyme from the group of acid hydrolases, which is found in polymorph nuclear leukocytes, myelocytes, plasma cells, megakaryocytes, platelets, and lymphocytes as well as mononuclear phagocytic system cells, is mostly detected in the B lymphocyte population of poultry.^16^

In this study, it was aimed to investigate the possible embryotoxic effects of propofol, an IV anaesthetic agent that is frequently preferred in anaesthesia practice, on peripheral blood lymphocytes by using enzyme histochemical methods.

## Methods

### Egg Material

In the study, 430 hatching eggs belonging to the commercial white layer named ATABEY produced by the Turkish Ministry of Food, Agriculture, and Livestock Poultry Research Institute were used as material. The study was carried out with the approval of the Ethics Committee of Selçuk University, Faculty of Veterinary Medicine, Experimental Animal Production and Research Centre, dated 29 March 2016 and numbered 2016/40.

### Propofol

A 2% propofol (Fresenius Kabi, Austria) IV injectable solution was used in this study.

### Preparation of Propofol Solutions

Propofol is used at a dose of 2.5 mg kg^−1^ in humans for the induction of general anaesthesia in clinical use. In this study, 2.5 mg kg^−1^, 12.5 mg kg^−1^, and 37.5 mg kg^−1^ doses of propofol were administered to eggs, each weighing an average of 50 g. The solution was diluted with 0.9% isotonic NaCl and the volume was standardised to 100 μL for each dose option.

### Design of Experimental Groups and Injection of Propofol into Eggs

In this study, the eggs were divided into 5 groups. According to the experience obtained from our previous embryotoxic studies, because it was foreseen that the toxic effects of high doses of the applied chemicals would be high, the number of chicks that would hatch alive would decrease in a dose-dependent manner. So, the number of eggs used in the groups was increased depending on the dose.

Control group (55 eggs): No treatment was applied to these eggs in this group.

Solvent-control group (60 eggs): These eggs were given 0.9% NaCl in a volume of 100 µL via the air chamber. 

2.5 mg kg^−1^ propofol group (90 eggs): 2% Propofol at a dose of 2.5 mg kg^−1^ (6.25 µL 2% propofol + 93.75 µL 0.9% NaCl) was given to eggs in a volume of 100 µL via the air chamber.

12.5 mg kg^−1^ propofol group (100 eggs): 2% Propofol at a dose of 12.5 mg kg^−1^ (31.25 µL 2% propofol + 68.75 µL 0.9% NaCl) was given to eggs in a volume of 100 µL via the air chamber.

37.5 mg kg^−1^ propofol group (125 eggs): 2% Propofol at a dose of 37.5 mg kg^−1^ (93.75 µL 2% propofol + 6.25 µL 0.9% NaCl) was given to eggs in a volume of 100 µL via the air chamber.

The blunt ends of the eggs, except for the control group, were wiped with 96% ethanol for disinfection before injection and pierced with an egg drill. All injection procedures were performed under sterile conditions in a laminar flow cabinet, with a sterile tipped micropipette, at 100 μL per egg volume and through the air chamber of the eggs. The holes were closed with liquid paraffin immediately after the injections. Subsequently, the eggs were placed into an incubator at 37.5°C, at 65% relative humidity, by turning 180° once every 2 hours in the incubator in the Histology and Embryology Department of the Faculty of Veterinary Medicine of our university.

### Blood Samples

On the 21st day of incubation, blood samples were taken from 6 chicks from each group. A total of 4 smears were prepared from each of the blood samples, 2 for ANAE and 2 for ACP-ase enzyme demonstrations. The smears were fixed in glutaraldehyde–acetone fixation solution (pH = 4.8) at −10°C for 3 minutes. At the end of these periods, the smears were washed 3 times with distilled water and dried at room temperature.^17^

### Alpha-Naphthyl Acetate Esterase Demonstration

For this purpose, 20 mg of a substrate (alpha-naphthyl acetate, N-8505-Sigma) dissolved in 0.8 mL of acetone (Merck) was slowly dropped into 80 mL of buffered phosphate solution with a pH of 5.0. Then, 4.8 mL of hexazotised pararozaniline solution obtained by mixing 2.4 mL of 4% sodium nitrite (S-3421, Merck) solution and 2.4 mL of pararozaniline (P-3750, Merck) solution for 2 minutes was added to the buffered phosphate solution containing substrate. After the pH of the prepared solution was adjusted to 5.8 with 1 N NaOH solution, it was filtered.^17^

### Acid Phosphatase Demonstration

For this purpose, buffered Michael's Veronal-acetate solution at pH 5.0 and 30 mg of Naphthol AS-BI phosphate (N-2125, Sigma) dissolved in 3 mL of N, N-dimethylformamide were used as substrate. Three millilitres of the substrate solution added to 15 mL of the buffer solution were mixed with 39 mL of distilled water, then 4.8 mL of hexazotised (2.4 mL of pararozaniline, 2.4 mL of 4% sodium nitrite) pararozaniline solution was added. After the final pH of the mixture was adjusted to 5.0 with 1 N NaOH solution, it was filtered.^17^

**Figure 1.**
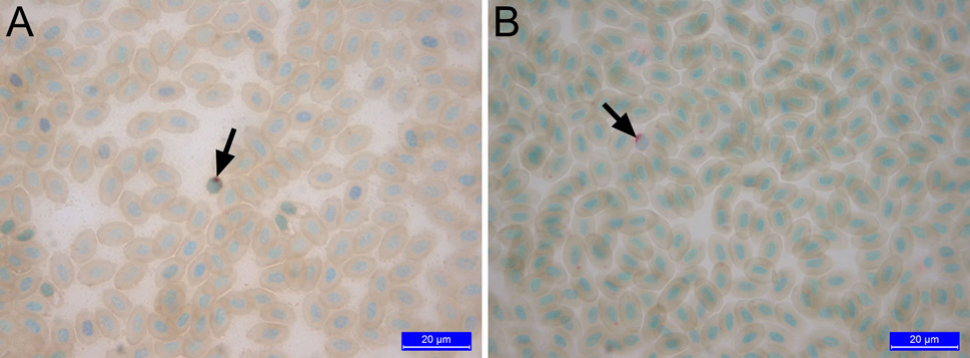
Peripheral blood alpha naphthyl acetate esterase (A) and acid phosphatase (B)-positive lymphocytes. Arrows: Enzyme-positive lymphocytes.

Blood smears were kept in the prepared incubation solutions at 37°C for 1 hour in a controlled manner. After the appearance of reddish-brown granules for the ANAE enzyme (Figure 1A) and pinkish-red granules for the ACP-ase enzyme (Figure 1B), the incubation processes were terminated and the smears were washed 3 times with distilled water and 1% methyl-green dye was applied. In both blood smears, the cells with lymphocyte morphology and having l1-3 granules were considered enzyme positive. Positive lymphocyte ratios were determined by counting a total of 200 lymphocytes in each of the blood smears.

### Statistical Analyses

Positivity ratios were analysed using the Angle (Arc Sinus) transformation method.^18^ According to this method, a 1-way analysis of variance was performed on the transformed data. Tukey test was used to compare the parameters of the groups with each other. Statistical Package for Social Sciences version 15.0 (SPSS Inc.; Chicago, IL, USA) package program was used for the statistical evaluation of all data. The criterion of statistically significant for all data was at *P* < .05.

## Results

**Alpha Naphthyl Acetate Esterase Enzyme Results in Peripheral Blood Lymphocytes**

Peripheral blood ANAE-positive lymphocyte rates of the groups are given in Table 1. Alpha naphthyl acetate esterase-positive lymphocyte ratios obtained from the chickens of the control and solvent-control groups were very close to each other, and no statistically significant difference was found between them. However, when compared with the control and solvent-control groups, significant decreases were observed in the peripheral blood ANAE-positive lymphocyte ratios of the chicks from the propofol-injected groups, which were also found to be statistically significant (*P* < .05). While the difference between 2.5 mg kg^−1^ and 12.5 mg kg^−1^ propofol groups was not significant, the difference between these 2 groups and the 37.5 mg kg^−1^ propofol group was found to be significant (*P* < .05).

### Acid Phosphatase Enzyme Results in Peripheral Blood Lymphocytes

Peripheral blood ACP-ase-positive lymphocyte rates of the groups are given in Table 1. It was found that the results were similar to the results of the ANAE enzyme. Accordingly, ACP-ase-positive lymphocyte ratios obtained from the chickens of the control and solvent-control groups were very close to each other, and the difference between them was not statistically significant. On the other hand, peripheral blood ACP-ase-positive lymphocyte ratios of the chicks in the propofol-injected groups were found to be quite low compared to the control and solvent-control groups, and the difference between them was statistically significant (*P* < .05). While the results obtained from the 2.5 mg kg^−1^ and 12.5 mg kg^−1^ propofol groups were similar to each other, the difference between these 2 groups and the 37.5 mg kg^−1^ propofol group was significant (*P* < .05).

## Discussion

Most of the studies on the possible side effects of propofol have focused on individuals exposed to the application. However, studies on the effects of propofol exposure on the embryo/foetus are very limited. It has been determined that 0.5%-2% of pregnant women are exposed to general anaesthesia for non-obstetric surgery every year in the USA, and it has been reported that this rate does not include women who are not yet aware of their pregnancy at the time of surgery.^19^ It is stated that propofol can potentially cause similar side effects in newborns due to its rapid passage through the placenta. Neurological and adaptive capacity scores are found to be lower in individuals born to mothers exposed to high-dose and/or long-term propofol before birth.^20,21^ The weakness of neurological reflexes, which reveals some motor skills, of the offspring born from rats exposed to different doses of propofol on the 18th day of pregnancy and followed up for 28 days postnatally, revealed that propofol administered to the mothers during pregnancy adversely affected the development of the offspring born from these mothers.^22^ Similarly, it has been suggested that neuron loss increases, synaptophysin levels decrease in the hippocampal region, and permanent learning disabilities occur due to increased caspase-3 levels in the brains of baby rats of rats administered IV propofol on the 18th day of pregnancy.^23^ For this reason, it is recommended that women of childbearing age should have pregnancy control before surgical procedures that require anaesthesia.

Nowadays, newly discovered molecules or combinations have to pass through many in vivo and in vitro test environments to determine possible toxicity, embryotoxicity, and side effects before they can be used in routine use in humans and animals. It is an extremely important advantage that the applications made on animal models to be used in this process can be performed even in average laboratory conditions, with the fastest possible result, and low cost.^13^ The CHEST, which has the highest level of these advantages and uses fertile chicken eggs, is a material that has significant advantages with its low cost, easy access, and the use of a sufficient number of fertile eggs in terms of statistical evaluations. At the same time, studies on chicken eggs comply with ethical rules and legal restrictions as well as animal rights, as they minimise the pain to be caused to a living organism. In addition, the results obtained can be adapted to mammals.^12^

Lymphocytes, which have a vital role in protecting the body against infections, are divided into 2 groups as B and T lymphocytes. B lymphocytes, which pass from the peripheral circulation to tissues with antigenic stimulation and differentiate into antibody-producing plasma cells, develop in the bone marrow, and T lymphocytes, which are responsible for cellular immunity, develop in the thymus.^24^ Enzyme histochemical staining, which is used to distinguish these cells from each other at the light microscopy level, can be preferred to more precise but costly methods because they can be performed even under simple laboratory conditions, have low costs, give results in a short time, and are reproducible. It has been reported that ANAE positivity detected in lymphocytes is characteristic of T lymphocytes of some mammalian and poultry species, while ACP-ase positivity is suggested to be specific for B lymphocytes in chickens. Peripheral blood ANAE and ACP-ase-positive lymphocyte ratios, which have clinical value because they are used in the differential diagnosis of some diseases, are also important in terms of allowing the monitoring of the immunological status of the examined species in studies investigating the effects of certain chemicals or physiological processes such as pregnancy on the immune system.^16,25^

In this study, the effect of different doses of propofol given to fertile chicken eggs via the air chamber just before incubation was investigated on the peripheral blood ANAE and ACP-ase-positive lymphocyte rates of the chicks hatched from these eggs. As a result of the light microscopic evaluations, it was determined that the peripheral blood ANAE and ACP-ase-positive lymphocyte ratios of the chicks hatched from the eggs treated with propofol decreased significantly when compared to the control group without any treatment and the solvent-control group eggs with only saline injection (Table I). Chikutei et al^26^ have reported that propofol, which has a neuroprotective effect against oxidative stress, increases cell death caused by H_2_O_2_ in rat thymocytes and have suggested that caution should be exercised in its use since propofol has different effects on different cells. Because it is thought that the decrease in lymphocyte counts will increase the sensitivity to the risk of infection, and in addition to this risk in newborns, it is thought that it will bring along important problems such as not getting the desired result from the vaccination programs.

The limitation of this study may be the evaluation of chicks immediately after hatching. The chicks could be followed for a longer period of time and re-evaluated. Thus, it was possible to see how the lymphocyte levels of the groups would change over time.

## Conclusion

There is a possibility that women of childbearing age have surgery at a time when they are not yet aware of the pregnancy. In addition to being the most important period in terms of organogenesis, this period is also the period when the embryo is most sensitive to physical and chemical factors. Like all other organs and systems, the immune system can also be affected by the side effects of the agents exposed during this period, and tissue-specific adverse effects can be seen even in the most innocent agents. In this study, the negative effect of propofol evaluated in the study on peripheral blood T and B lymphocytes with increasing doses in a dose-dependent manner has been clearly demonstrated. We think that it is very important to consider these factors in anaesthesia management and to choose the method and agent accordingly.

## Figures and Tables

**Table 1.** Peripheral Blood ANAE and ACP-ase-Positive Lymphocyte Rates

Groups (n = 6)	ANAE (%) (X ± SD)	ACP-ase (%) (X ± SD)
Control group	38.83 ± 3.12^a^	63.91 ± 6.06^a^
Solvent-control group	36.41 ± 2.76^a^	64.83 ± 3.71^a^
2.5 mg kg^−1^ propofol group	28.75 ± 3.02^b^	42.25 ± 4.68^b^
12.5 mg kg^−1^ propofol group	29.16 ± 2.38^b^	43.16 ± 4.03^b^
37.5 mg kg^−1^ propofol group	21.91 ± 2.57^c^	32.75 ± 6.66^c^
F value	35 084	42 699
*P*	.000	.000

ACP-ase, acid phosphatase; ANAE, alpha naphthyl acetate esterase; SD, standard deviation.

^a–c^Differences between values with different letters in the same column have statistical significance.

*P* < .05.
